# Comparison of quantitative lung ultrasound scores with automated quantitative CT: to overcome the ultrasound limitations

**DOI:** 10.1093/rheumatology/keaf328

**Published:** 2025-06-12

**Authors:** Davide Mohammad Reza Beigi, Greta Pellegrino, Nicholas Landini, Marco Emanuele Diana, Gregorino Paone, Ilaria Bisconti, Francesca Romana Di Ciommo, Marius Cadar, Elena Platania, Jacopo Landro, Simona Truglia, Valeria Panebianco, Fabrizio Conti, Valeria Riccieri

**Affiliations:** Dipartimento di Scienze Cliniche Internistiche, Anestesiologiche e Cardiovascolari, Sapienza University of Rome, Rome, Italy; Rheumatology Clinic ‘Madonna dello Scoglio’, Cotronei, Italy; IRCCS Ospedale Galeazzi – Sant’Ambrogio, Milan, Italy; Dipartimento di Scienze Biomediche e Cliniche, Università degli Studi di Milano, Milan, Italy; Dipartimento di Scienze Radiologiche, Oncologiche e Anatomo-Patologiche, Sapienza, University of Rome, Rome, Italy; Dipartimento di Scienze Radiologiche, Oncologiche e Anatomo-Patologiche, Sapienza, University of Rome, Rome, Italy; Dipartimento di Scienze Cardiovascolari e Respiratorie, Sapienze, University of Rome, Rome, Italy; Dipartimento di Scienze Cliniche Internistiche, Anestesiologiche e Cardiovascolari, Sapienza University of Rome, Rome, Italy; Dipartimento di Scienze Cliniche Internistiche, Anestesiologiche e Cardiovascolari, Sapienza University of Rome, Rome, Italy; Dipartimento di Scienze Cliniche Internistiche, Anestesiologiche e Cardiovascolari, Sapienza University of Rome, Rome, Italy; Dipartimento di Scienze Cliniche Internistiche, Anestesiologiche e Cardiovascolari, Sapienza University of Rome, Rome, Italy; Dipartimento di Scienze Cliniche Internistiche, Anestesiologiche e Cardiovascolari, Sapienza University of Rome, Rome, Italy; Dipartimento di Scienze Cliniche Internistiche, Anestesiologiche e Cardiovascolari, Sapienza University of Rome, Rome, Italy; Dipartimento di Scienze Radiologiche, Oncologiche e Anatomo-Patologiche, Sapienza, University of Rome, Rome, Italy; Dipartimento di Scienze Cliniche Internistiche, Anestesiologiche e Cardiovascolari, Sapienza University of Rome, Rome, Italy; Dipartimento di Scienze Cliniche Internistiche, Anestesiologiche e Cardiovascolari, Sapienza University of Rome, Rome, Italy

**Keywords:** interstitial lung disease, lung ultrasound, systemic sclerosis, quantitative chest tomography, lung texture analysis

## Abstract

**Objectives:**

Lung ultrasound (LUS) is emerging as a valuable tool for assessing systemic sclerosis-associated interstitial lung disease (SSc-ILD), although it traditionally explores only superficial lung regions. Building upon our preliminary findings, this study investigated correlations between quantitative LUS scores and automated quantitative computed tomography (qCT) measurement of ILD extent, including both superficial and deeper lung involvement.

**Methods:**

Between 2021 and 2023, 82 consecutive SSc patients underwent concurrent LUS and CT scans. Total B-lines (BL) count (range 0–140) and our novel pleural line irregularity (PLI) score (range 0–28) were obtained using a 14-intercostal space scanning protocol. CT scans were analysed by automated texture analysis software, quantifying volumes of ILD, ground-glass opacities (GGO) and reticulations (RET), segmented in three levels (apices, midfields, bases) and subdivided in surface and core lung parenchyma.

**Results:**

Total BL count and PLI score correlated with total ILD, GGO and RET volumes (all *P* < 0.0001), as well as with surface and core ILD volumes (all *P* < 0.0001). Basal lung BLs and PLI score correlated with basal ILD, GGO, RET (all *P* < 0.005) and corresponding surface and core ILD volumes (all *P* < 0.005). Mid-lung PLI correlated also with corresponding ILD-related changes and surface and core ILD (all *P* < 0.005). These associations were confirmed by multivariate regression analysis.

**Conclusions:**

Quantitative LUS score correlated with qCT-defined ILD extent, especially at lung bases. LUS scores (particularly the novel PLI score) were found to correlate with deeper ILD volume, suggesting potential to overcome traditional LUS limitations related to superficial lung assessment.

Rheumatology key messages

- Quantitative lung ultrasound scores correlate with automated quantitative CT findings in systemic sclerosis ILD.
- Lung ultrasound scores correlate with deeper lung involvement, particularly at lung bases in SSc-ILD.
- The novel pleural line irregularity score confirms its validity in SSc-ILD assessment.

## Introduction

Systemic sclerosis (SSc) is a chronic complex disease characterized by immune system disturbances, vascular changes and fibrosis [1]. Interstitial lung disease (ILD) is one of the most frequent organ manifestations of the disease, with a high impact on patient morbidity and mortality [2, 3]. Interest in this condition has soared in recent years, with the development of dedicated international guidelines for diagnosis and management [4, 5]. The cornerstones for diagnosis and monitoring of SSc-ILD are represented by chest computed tomography (CT) in combination with pulmonary function tests (PFTs) [6], although the variability of onset, severity and progression of the disease make the management of SSc-ILD challenging [7]. Moreover, CT implies a radiation exposure with a biological risk for patients, especially if lifelong follow-up are needed: in this context, complementary radiation-free tools such as lung ultrasound (LUS) and magnetic resonance imaging are gaining ground [8, 9].

LUS has emerged in recent years as an easy accessible and valuable method for the investigation of SSc-ILD [10, 11]. It showed high accuracy in identifying SSc-ILD compared with the gold standard of CT as well as significant association with PFT parameters [12–15]. Lately, the role of LUS has also emerged in the assessment of disease progression and treatment response [10, 16]. Key LUS findings are represented by the sonographic artifacts of B-lines (BLs) and pleural line irregularity (PLI), the definition of which has recently undergone standardization by the Outcome Measures in Rheumatology (OMERACT) Ultrasound Working Group [17]. Several scanning approaches have been proposed over the years. The 14 lung intercostal spaces assessments (LIS) proposed by Gutierrez *et al.* were shown to be reliable compared with more time-consuming approaches evaluating a wider number of LIS [18, 19]. This method has been used in the development of SSc-ILD interpretation criteria based on PLI [20] and for detection and monitoring of subclinical SSc-ILD [21]. However, most published LUS scoring systems for SSc-ILD diagnosis and severity assessment focus on BLs [14, 16, 22]. Recently, our group developed a quantitative PLI score using a 14-LIS protocol. In a preliminary work, this score showed an anatomical correlation with SSc-ILD extent on three-levels automated quantitative chest tomography (qCT) [23]. Despite increasingly solidifying evidence, a defining role of LUS in SSc-ILD is still lacking, also due to the intrinsic limitations of the US method compared with CT, including the possibility to explore only superficial areas of the lung parenchyma [24].

Based on previous assumptions, the purpose of the present study was to confirm the results of our preliminary study [23] and investigate a possible association of quantitative LUS scores with ILD of the lung surface and core assessed by qCT. Hence, the primary outcomes were to assess the correlation between the total number of BLs and the total PLI score with the volume of ILD, as well as with the ILD of surface and core lung as detected by qCT. As secondary outcomes, the correlation of number of BLs and PLI score with the volume of different qCT findings at three-levels qCT (lung apices, midfields and bases) analysis was evaluated.

## Materials and methods

During 2021–2023, consecutive adult patients affected by SSc according to 2013 ACR/EULAR classification criteria referring to the Scleroderma Clinic of ‘Policlinico Umberto I’ (Rome, Italy) who underwent CT were evaluated for enrolment. Exclusion criteria were the presence of pulmonary arterial hypertension (either known in history or detected by echo signs), lower airway infections in the previous six months, history of chest radiotherapy and inadequate CT image quality (movement artifacts, pleural effusion, alterations other than ILD). Selected patients underwent LUS on the same day of CT. PFTs were carried out within a month. LUS was performed by two certified operators (G.P. and D.M.R.B.), blinded to each other and to CT, using an Esaote^®^ Mylab™ Gamma (Genoa, Italy) equipped with a 3–13 MHz linear probe. The 14-LIS scanning method was used and LIS were divided for each lung into apices (II parasternal), midfields (IV mid-clavear, IV anterior and median axillary) and lung bases (VIII posterior axillary, sub-scapular and paravertebral) as previously described [23]. Total BLs number was collected (from 0 to 140) and the PLI score proposed by our centre was applied (from 0 to 28) [23]. CT scans were performed with adequate parameters, reconstruction and windowing to be visually and automatically assessed. Two thoracic radiologists, by consensus, determined the presence of ≥10% ILD in included images, as well as the pattern [e.g. usual interstitial pneumonia (UIP), non-specific interstitial pneumonia (NSIP) or organizing pneumonia (OP)] [25]. All included CT were analysed by Imbio^®^ Lung Texture Analysis™ software based on Computer-Aided Lung Informatics for Pathology Evaluation and Rating (CALIPER) technology, in order to identify and quantify the volume (cm^3^) of healthy lung, ground-glass (GGO), reticulations (RET), honeycombing (HC), total fibrosis (RET + HC) and total ILD (GGO + RET + HC). The software further divided lung parenchyma into ‘surface’ and ‘core’, each representing about 50% of lung volume. Clinical laboratory data were also collected.

Written informed consent was obtained from every patient enrolled in the study. The ethics committee of Policlinico Umberto I hospital (Comitato Etico Territoriale Lazio Area 1) approved the study (Rif. 7284 Prot. 0681/2023).

## Statistical analysis

Continuous variables were expressed as means and standard deviations (SD) or medians and interquartile ranges (IQR) for non-normally distributed data. The distribution of continuous variables was evaluated with the Shapiro Wilks test. Comparisons between continuous variables were performed with the Mann–Whitney *U* test or the *t* test where appropriate. Categorical variables were expressed as numbers and percentages and compared using the χ^2^ test or Fisher test, where appropriate. To assess the correlation between continuous variables, we used Kendall’s tau-b (τ) correlation coefficient. This non-parametric test was chosen for the distribution characteristics and low sample size of variables, other than its robustness in handling ties [26].

A multivariate linear regression analysis was performed to assess the independent association between the BLs and PLI scores and the volume of different pathological tissue at qCT, adjusted for multiple covariates. Covariates included in the model were selected when they emerged as confounder at the univariate comparison analysis. Prior to analysis, assumption of linearity, homoscedasticity, multicollinearity and normality of residuals were assessed to ensure the validity of the model. When the linearity assumption was not confirmed, a log-transformation of variable values was performed. The results of final models were reported as regression coefficient (β) with standard error (SE) and corresponding *P*-values. All statistical analyses were performed using R Studio graphical interface version 2023.12.1 + 402 for R software environment v. 4.3.0. All tests were two-sided with a significance level set at *P* < 0.05.

## Results

### Patients’ characteristics

In total, 82 patients were enrolled (72 females, 10 males), with a median age (quartiles) of 63 years (53; 72) and a median disease duration of 7.5 years (3; 14). Median ILD duration from CT diagnosis was 4 (2; 8) years. Forty-five patients (55%) were on immunosuppressant therapy. Median predicted FVC% and DLCO% values were, respectively, 96 (77; 110) and 72 (58; 84).

Characteristics of the study population are reported in Table 1.

**Table 1.** Characteristics of study population and descriptive analysis of lung ultrasound (LUS) and automated quantitative computed tomography (qCT) assessment

Female/male, N (%)	72/10 (86.2/13.8)
Median age, years (quartiles)	63 (53; 72)
Median disease duration, years (quartiles)	7.5 (3; 14)
Diffuse/limited cutaneous disease form, N (%)	38/44 (46/54)
ILD ≥10% on CT, N (%)	36 (44)
Smoking (current or ever), N (%)	27 (33)
Anti-topoisomerase I antibodies positivity, N (%)	33 (40)
Anti-centromere antibodies positivity, N (%)	24 (29)
Digital ulcer presence, N (%)	23 (28)
Puffy fingers, N (%)	29 (35)
Sclerodactyly, N (%)	46 (56)
Median ILD duration, years (quartiles)	4 (2; 8)
Diffuse/limited cutaneous disease form, N (%)	38/34 (46.3/41.4)
Immunosuppressant therapy, N (%)	45 (55)
FEV1 %, median (quartiles)	94 (75; 107)
FVC %, median (quartiles)	96 (77; 110)
TLC%, median (quartiles)	88 (73; 100)
DLCO %, median (quartiles)	72 (58; 84)
B-lines no. (0–140), median (quartiles)	16.5 (6.25; 44.5)
PLI score (0–28), median (quartiles)	11 (5; 19.75)
qTC abnormality, cm^3^/%, median (quartiles)	
Healthy lung	3494/86 (2264/76; 4233/94)
Ground-glass (GGO)	53/2 (5.1/0.12; 180/6.6)
Reticulations (RET)	45/1 (21/0.44; 111/3.6)
Honeycombing (HC)	6.7/0.2 (1.6/0.037; 38/1)
Fibrosis (RET + HC)	67/1.8 (28/0.7; 197/5.7)
ILD (GGO + RET + HC)	232/5 (57/1.3; 478/15)
Lung surface ILD	168 (39; 371)
Lung core ILD	33 (10; 97)

DLCO: diffusing capacity of carbon monoxide; FEV1: forced expiratory volume in the 1st second; FVC: forced vital capacity; ILD: interstitial lung disease; PLI: pleural line irregularity; TLC: total lung capacity.

### LUS assessment

Total median BLs were 16.5 (6.25; 44.5) and median PLI score was 11 (5; 19.75) (Table 1). Apices, midfields and lung bases BLs medians were 1 (0; 4), 6.5 (1; 16) and 11 (3.26), respectively, whereas median PLI score was 1 (0; 2), 5 (2; 8) and 6 (2; 11) (Table 2).

**Table 2.** LUS and qCT 3-levels division, median (quartiles)

	Lung apices	Lung midfields	Lung bases
B-lines no. (0–140)	1 (0; 4)	6.5 (1; 16)	11 (3.26)
PLI score (0–28)	1 (0; 2)	5 (2; 8)	6 (2; 11)
qTC abnormality, cm^3^, median (quartiles)	Lung apices	Lung midfields	Lung bases
Ground-glass	0.2 (0.00; 5.4)	5.7 (0.67; 50)	31 (2.3; 117)
Reticulations	1.2 (0.1; 7.5)	10 (3.9; 32)	30 (8.9; 66)
Fibrosis	1.6 (0.15; 9)	4.6 (0.85; 13)	11 (2.4; 43)
ILD	12 (1.9; 45)	38 (9.9; 139)	148 (34; 284)
Lung core ILD	1.4 (0.04; 8.2)	13 (2.5; 39)	16 (2.2; 44)
Lung surface ILD	8.7 (1–6; 31)	27 (3.6; 86)	109 (20; 210)

LUS: lung ultrasound; qCT: automated quantitative computed tomography.

### CT and qCT analysis

Thirty-six patients (44%) had ILD ≥10% on CT, two of which had a UIP pattern and the rest a NSIP pattern.

At qCT analysis, GGO volume (cm^3^) was 53 (5.1; 180), RET was 45 (21; 111), HC 6.7 (1.6; 38), total fibrosis 67 (28; 197) and total ILD 232 (57; 478) (Table 1). Median lung surface ILD was (cm^3^) 168 (39; 371) and core ILD was 33 (10; 97) (Table 1). Apices, midfields and lung bases GGO, RET, total fibrosis and total ILD volumes, as well as surface and core lung division are reported in Table 2.

Surface lung ILD volume was significantly greater than that of core lung [median (IQR) 169 (37; 372) cm^3^  *vs* 33 (10; 85) cm^3^, *P* < 0.001]. The same emerged for GGO [60 (4.8; 236) *vs* 5 (0.4; 27), *P* < 0.001] and for RET 17 [(6.4; 51) *vs* 7.9 (1.3; 23), *P* < 0.004)] volumes.

### Correlations between LUS and qCT

#### Overall lung correlations

Both total BLs and PLI score were found to correlate negatively with healthy lung volume (respectively, τ −0.36, *P* < 0.014 and τ −0.5, *P* < 0.0001) and positively with total ILD volumes (τ 0.43, *P* < 0.0001 and τ 0.58, *P* < 0.0001), GGO (τ 0.35, *P* < 0.017 and τ 0.45, *P* < 0.0001), RET (τ 0.44, *P* < 0.0001 and τ 0.56, *P* < 0.0001) and total fibrosis (τ 0.44, *P* < 0.0001 and τ 0.51, *P* < 0.0001).

Both total BLs and PLI score were found to correlate with whole lung surface and core ILD (respectively, τ 0.46, *P* < 0.0001 and τ 0.29, *P* < 0.011 for BLs and τ 0.59, *P* < 0.0001 and τ 0.45, *P* < 0.0001 for PLI score).

### Three-levels qCT analysis

At 3-levels qCT analysis, BLs correlated at lung bases with both surface and core ILD (respectively, τ 0.44, *P* < 0.0002 and τ 0.36, *P* < 0.0031). PLI showed a correlation with surface ILD of apices (τ 0.25, *P* < 0.039), midfields (τ 0.46, *P* < 0.0001) and lung bases (τ 0.51, *P < *0.0001) and with core ILD of midfields (τ 0.38, *P* < 0.0015) and lung bases (τ 0.42, *P* < 0.0003) (Table 3).

**Table 3.** Kendall’s correlation with reported τ coefficient (CI) and *P*-value of surface and core ILD with three-levels qCT analysis

B-lines no.	Lung apices	Lung midfields	Lung bases
Core lung ILD	ns	ns	0.36 (0.12; 0.55), **0.0031**
Surface lung ILD	ns	ns	0.44 (0.22; 0.62), **0.0002**

PLI score	Lung apices	Lung midfields	Lung bases
Lung core ILD	ns	0.38 (0.15; 0.57), **0.0015**	0.42 (0.19; 0.6), **0.0003**
Lung surface ILD	0.25 (0.00; 0.47), **0.039**	0.46 (0.24; 0.64), **<0.0001**	0.51 (0.13; 0.67), <**0.0001**

Bold text indicates significant *P*-values.

CI: confidence interval; ILD: interstitial lung disease; qCT: automated quantitative computed tomography.

When a multivariate linear regression model was implemented to assess the association between LUS scores and core or surface ILD, an increase in BLs and PLI was independently associated with total surface ILD (Table 4).

**Table 4.** Results from multiple linear regression model of B-lines no. and PLI score with core ILD including surface ILD as covariate

B-lines no.	β coefficient	SE	*P*-value
Intercept	18.10	4.25	**<0.0001**
Surface ILD	0.04	0.02	**0.0187**
Core ILD	−0.02	0.03	0.5030

PLI score	β coefficient	SE	*P*-value
Intercept	10.83	1.02	**<0.0001**
Surface ILD	0.07	0.02	**0.00498**
Core ILD	0.04	0.03	0.24281

Bold text indicates significant *P*-values.

ILD: interstitial lung disease; SE: standard error.

**Figure 1.**
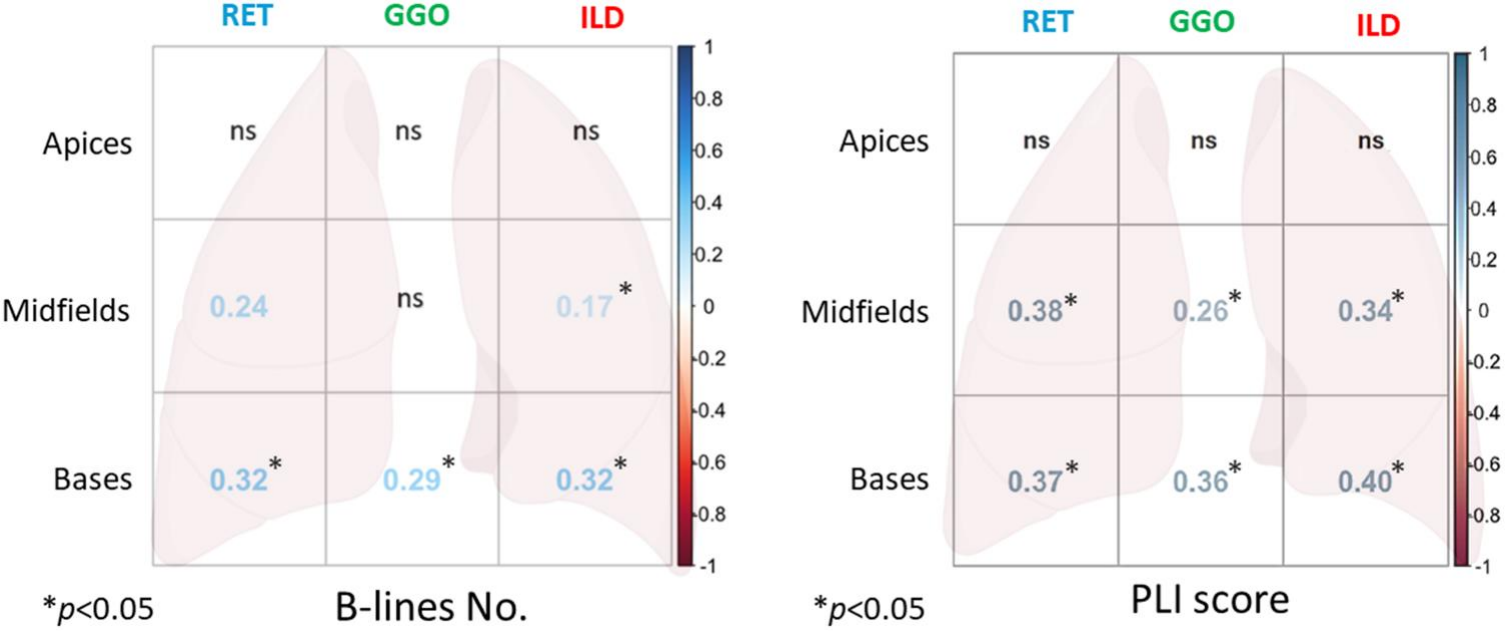
Correlogram showing the Kendall’s correlation with reported τ coefficient and *P*-values of B-lines and PLI score with three-levels qCT analysis. GGO: ground-glass opacities; PLI: pleural line irregularity; qCT: automated quantitative computed tomography; RET: reticulations

BLs of lung bases showed a correlation with ILD (τ 0.32, *P < *0.0001), GGO (τ 0.29, *P* < 0.002) and RET (τ 0.32, *P < *0.0001) volumes of lung bases at qCT. At lung midfields, BLs correlated with ILD (τ 0.17, *P* < 0.039) and RET volumes (τ 0.24, *P* < 0.0029) (Fig. 1A).

PLI score of both lung bases and midfields correlated with ILD (respectively, τ 0.4, *P < *0.0001 and τ 0.34, *P < *0.0001), GGO (τ 0.36, *P < *0.0001 and τ 0.26, *P* < 0.0015) and RET (τ 0.36, *P < *0.0001 and τ 0.37, *P < *0.0001) volumes of the same fields on qCT (Fig. 1B).

In multiple linear regression analysis, both basal lung BLs and PLI score were associated with basal ILD, and basal PLI score was also associated with basal GGO in a model that accounted for immunosuppressant use as a confounding factor. This association persisted even after adjusting for covariates such as age, disease duration, ILD duration and smoking (Table 5).

**Table 5.** Multiple linear regression model of basal lung ILD and GGO volumes with basal B-lines no. and basal PLI score including immunosuppressant therapy as covariate

Basal ILD volume	β coefficient	SE	*P*-value
Intercept	1.00	1.00	**<0.0001**
Basal lung B-lines	2.71	1.00	**<0.0001**
Immunosuppressant therapy	1.00	1.00	**0.0372**

Basal lung GGO volume	β coefficient	SE	*P*-value
Intercept	36.14	1.26	**<0.0001**
Basal lung B-lines	1.43	1.08	**<0.0001**
Immunosuppressant therapy	2.22	1.08	**0.0168**

Basal lung GGO volume	β coefficient	SE	*P*-value
Intercept	36.14	1.50	**<0.0001**
Basal lung B-lines	1.43	1.15	**0.000226**
Immunosuppressant therapy	2.22	1.74	0.299493

Bold text indicates significant *P*-values.

GGO: ground-glass opacities; ILD: interstitial lung disease; PLI: pleural line irregularity; SE: standard error.

Regarding PFTs values, both total BLs and PLI negatively correlated with predicted FEV1% (τ −0.27, *P < *0.0184 and τ −0.3, *P < *0.0063), FVC% (τ −0.33, *P < *0.0027 and τ −0.4, *P < *0.0003), DLCO% (τ −0.27, *P < *0.0159 and τ −0.4, *P < *0.0002) and TLC% (τ −0.35, *P < *0.0016 and τ −0.48, *P < *0.0001).

Additionally, comparing PFTs witch qCT, FVC% negatively correlated with ILD, GGO and RET volumes of both whole lung (*P < *0.0005) and apices, midfields and bases (*P < *0.05). On the other hand, DLCO% negatively correlated only with total ILD volume, but at three-levels analysis a negative correlation with ILD, GGO and RET volumes of apices, midfields and bases emerged (*P < *0.05). Finally, FVC% showed a negative correlation with overall surface and core ILD, GGO and RET volumes, whereas DLCO% negatively correlated with surface ILD, GGO and RET (*P < *0.05), but only with core lung GGO (*P < *0.0088).

## Discussion

The present study showed that quantitative LUS BLs and PLI score correlate with total ILD, GGO, RET and fibrosis volumes as identified by automated qCT. These results are consistent with our preliminary study and underscore the potential of LUS as a non-invasive tool for SSc-ILD assessment [23]. Moreover, as a novel finding, LUS scores were found to correlate with ILD of both the lung surface and core. In addition, these US scores were found to be associated with ILD and ILD-related changes volume on three-levels qCT, confirming again our preliminary results [23].

Our data showed that ILD, GGO and RET were prevalent at the periphery than in the core, in line with NSIP characteristics [25]. Both LUS scores correlated with total lung surface and core ILD. This finding is particularly relevant, as one of the known limitations of LUS in ILD evaluation is its restricted ability to assess deeper parenchymal areas. However, in the context of SSc-ILD, which often presents with NSIP pattern predominantly affecting subpleural regions, this limitation may be less impactful [25].

However, at three-levels qCT analysis, basal BLs were found to correlate with basal lung surface and core ILD, whereas PLI score at three-levels qCT correlated with the whole-lung surface ILD volume and with core ILD of lung bases and midfields. These results are open to several considerations. Firstly, it seems that both LUS scores, even if affected by the presence of surface ILD, are associated to the ILD of the lung core, and this aspect in our study is more evident at the level of lung bases, which are also the area most frequently affected by SSc-ILD. Beyond the periphery, SSc-ILD presenting as NSIP is usually predominant at lung bases [25], and accordingly, LUS scores focusing on lung bases assessment have been proposed [22]. Therefore, following the natural course of ILD, an increase of the extent of ILD would lead to an increase in the severity at lung bases, which correspond to a wider involvement of the core. Hence, both BLs and PLI LUS scores at lung bases could also be used as a surrogate for core ILD detection. This would somehow allow for the traditional limitation of LUS to explore only the superficial parenchyma to be overcome and give more opportunities for LUS use in the assessment of SSc-ILD. Additionally, these results further appraise the utility of PLI for a comprehensive LUS assessment of SSc-ILD. As mentioned above, in fact, most of the current ultrasound scores are based on B-line evaluation. On the other hand, the role of PLI in the assessment of LUS is becoming more established [17]. Indeed, a reduction of PLI score was found to occur during nintedanib therapy in SSc-ILD patients [16].

Moreover, a negative correlation with PFTs values and both LUS score emerged, in agreement with literature data [12]. These data further reinforce the role of the PLI score we proposed in the LUS assessment of SSc-ILD [23], and the utility of the simplified 14-scans assessment method proposed by Gutierrez and colleagues [18]: this confirms that LUS could be a valid instrument to justify functional worsening with lung parenchymal abnormalities, adding useful and alternative data to address patients’ management.

Finally, our results show a correlation of qCT analysis with PFTs values, particularly FVC%, confirming the reliability of qCT in reflecting lung function [27, 28]. In addition, FVC% values were found to be associated with ILD and ILD-related changes volume in the lung core, suggesting that these structural changes may have an influence on functional aspect.

Limitations of this study are the relatively small sample size and it single-centre design. Moreover, although the percentage of patients with ILD is representative of the prevalence of the complication, some selection bias is present, as patients who had to have CT scans were selected, a proportion of whom did so for follow-up of ILD, thus increasing the pre-test probability of ILD presence. Our results should certainly be confirmed by other independent scenarios. The present study did not evaluate the sensitivity of LUS scores to temporal changes in ILD progression or treatment response over time, which remains to be established in future longitudinal studies. Furthermore, a valuable topic for future research would be the comparison of operator time and resource utilization between the two diagnostic tools used, which could not be assessed within the scope of the present study.

In conclusion, this study shows a correlation between LUS findings with ILD and lung fields ILD at qCT analysis, confirming and expanding our preliminary results. Additionally, for the first time, LUS findings (especially the PLI score proposed by our group) were found to correlate also with core lung ILD volume, suggesting the possibility of overcoming the traditional LUS limits to explore only superficial lung parenchyma.

## Data Availability

The data underlying this article will be shared on reasonable request to the corresponding author.
